# Poster Session I - A143 ANTIPLATELET THERAPY FOR THE PREVENTION OF GI DISEASE: AN UMBRELLA REVIEW

**DOI:** 10.1093/jcag/gwaf042.143

**Published:** 2026-02-13

**Authors:** D R Kim, A Gupta

**Affiliations:** Medicine, Queen’s University, Kingston, ON, Canada; University of Calgary, Calgary, AB, Canada

## Abstract

**Background:**

Gastrointestinal (GI) diseases are a leading global cause of morbidity and mortality, accounting for over 8 million deaths and 277 million disability-adjusted life years annually. Preventive measures have halved disease incidence over recent decades, underscoring their importance in reducing population burden. One potential intervention is antiplatelet therapy (APT), particularly aspirin. The cyclooxygenase-2 (COX-2) pathways are implicated in chronic inflammation and tumorigenesis, and prior evidence suggests aspirin may reduce cancer incidence and progression of liver fibrosis. However, existing meta-analyses examine diseases in isolation, exclude non-malignant conditions, and pool heterogeneous or unadjusted effect sizes.

**Aims:**

To comprehensively review and qualify the credibility of evidence regarding APT and the prevention of GI diseases, including malignant, premalignant, and non-malignant conditions.

**Methods:**

We conducted an umbrella review according to PRIOR and PRISMA guidelines. We systematically searched PubMed, Embase, and Medline from inception to September 5, 2025, for meta-analyses of randomized or observational studies reporting age- and sex- adjusted effect sizes (OR, RR, HR) linking APT and GI diseases. Risk of bias was assessed with AMSTAR-2 and NOS tools. All meta-analyses were re-analyzed using random-effects models to calculate standardized pooled effect sizes with 95% confidence intervals (95%CI). Evidence credibility was graded as convincing, highly suggestive, suggestive, or weak.

**Results:**

Eight eligible meta-analyses were included, encompassing 39 unique primary studies and 13 299 962 participants, of whom 4 292 084 (32.4%) were regular APT users. A convincing association was found between aspirin use and reduced odds of esophageal cancer (OR 0.55, 95%CI 0.46–0.66, p < 0.001, k = 7, n = 11 811). Suggestive evidence was identified for gastric cancer (OR 0.70, 95%CI 0.57–0.87, p < 0.001, k = 8, n = 3 020 649). Weak evidence supported reduced risk of hepatocellular carcinoma (HR 0.52, 95%CI 0.34–0.78, p = 0.002, k = 8, n = 2 067 882), and reduced recurrence of colorectal adenomas (RR 0.79, 95%CI 0.64–0.97, p = 0.02, k = 5, n = 1 991).

**Conclusions:**

In conclusion, our umbrella review underscores APT’s role in preventing GI disease. Namely, aspirin may have a potential role in preventing esophageal cancer and Barrett’s Esophagus through COX-2 inhibition. Diseases including pancreatic cancer and HCC have a potential benefit from aspirin; however, variations in adjustment protocols for current studies likely confound aspirin’s true effect in these diseases and do not reflect their multifactorial pathogenesis, extending beyond inflammation. Our review, while comprehensive, highlights the need for standardized adjustment protocols, updated randomized and observational studies, and expansion to include non-aspirin anti-inflammatory agents and other antiplatelet agents.

**Funding Agencies:**

NoneNA

